# An analysis of food and beverage advertising on bus shelters in a deprived area of Northern England

**DOI:** 10.1017/S1368980021005048

**Published:** 2022-07

**Authors:** Amy Heather Finlay, Scott Lloyd, Amelia Lake, Thomas Armstrong, Mark Fishpool, Mark Green, Helen J Moore, Claire O’Malley, Emma J Boyland

**Affiliations:** 1 Department of Psychology, University of Liverpool, Liverpool L69 7ZA, UK; 2 Public Health South Tees, Middlesbrough, UK; 3 School of Health & Life Sciences, Teesside University and Fuse – Centre for Translational Research in Public Health, London, UK; 4 SHLS Allied Health Professions Centre for Public Health, Teesside University, and Fuse – Centre for Translational Research in Public Health, London, UK; 5 SHLS Allied Health Professions Centre for Public Health, Teesside University, Middlesbrough, UK; 6 Middlesbrough Environment City, Middlesbrough, UK; 7 Department of Geography and Planning, University of Liverpool, Liverpool, UK

**Keywords:** HFSS, Outdoor, Advertising, Transport, Food, Less healthy

## Abstract

**Objective:**

To quantify the extent of food and beverage advertising on bus shelters in a deprived area of the UK, to identify the healthfulness of advertised products, and any differences by level of deprivation. The study also sought to assess the creative strategies used and extent of appeal to young people.

**Design:**

Images of bus shelter advertisements were collected via in person photography (in 2019) and Google Street View (photos recorded in 2018). Food and beverage advertisements were grouped into one of seventeen food categories and classified as healthy/less healthy using the UK Nutrient Profile Model. The deprivation level of the advertisement location was identified using the UK Index of Multiple Deprivation.

**Setting:**

Middlesbrough and Redcar and Cleveland in South Teesside.

**Participants:**

N/A

**Results:**

Eight hundred and thirty-two advertisements were identified, almost half (48·9 %) of which were for foods or beverages. Of food and non-alcoholic beverage adverts, 35·1 % were less healthy. Most food advertisements (98·9 %) used at least one of the persuasive creative strategies. Food advertisements were found to be of appeal to children under 18 years of age (71·9 %). No differences in healthiness of advertised foods were found by level of deprivation.

**Conclusions:**

Food advertising is extensive on bus shelters in parts of the UK, and a substantial proportion of this advertising is classified as less healthy and would not be permitted to be advertised around television programming for children. Bus shelter advertising should be considered part of the UK policy deliberations around restricting less healthy food marketing exposure.

Over the last several decades, the global food environment has become increasingly obesogenic^(1)^. The obesogenic environment is characterised by a global food system that produces more processed, affordable and effectively marketed food than ever before, interacting with local environmental factors to determine obesity prevalence^(2)^. In the UK, 63 % of adults are living with overweight or obesity, as are one-third of primary school leavers (children aged 11 years)^(3)^. Evidence suggests that obesity prevalence is disproportionately higher in those of lower socio-economic status (SES) compared with less deprived groups, although to date this is only in high-income countries^(4)^. One key aspect contributing to the obesogenic environment is food and non-alcoholic beverage marketing.

Evidence demonstrates that exposure to food and non-alcoholic beverage (hereafter: food) advertising can influence awareness, attitudes and preferences, purchase intent, purchase requests, purchase, and consumption, particularly in children^(5,6)^ and has been implicated in the aetiology of childhood obesity^(7,8)^. The food advertising to which children are exposed is often made more powerful by the use of persuasive creative strategies such as promotional characters and premium offers^(9)^. Most research documenting the immediate impact of food advertising on eating behaviour focuses on children who are thought to be more susceptible to its persuasive effects^(10)^. However, emerging evidence suggests that adolescents show long-term behavioural effects in response to food marketing^(11)^ and may be even more vulnerable than young children^(12)^. Adults can also be influenced by food marketing in ways that are detrimental to dietary health, such as changes in meal patterning (e.g. increased snacking) and dietary displacement^(13,14)^.

Research shows that consumers from more deprived or ethnic minority backgrounds are disproportionately exposed to unhealthy food advertising. A systematic review by Backholer *et al.* (2021)^(15)^ found that potential exposure to, and impact of, unhealthy food advertising was greater among ethnic minority groups and children from lower SES backgrounds. These inequalities in exposure to unhealthy food advertising are likely to add to the stark existing inequalities in health for those of lower SES in high-income countries^(4)^.

Most monitoring research to date has sought to quantify the extent and nature of food advertising on television^(16)^ and, to a lesser extent, in digital media^(17)^. Far less is known about outdoor advertising, even though it is estimated that 98 % of the UK population see outdoor advertising daily^(18)^ and UK outdoor advertising expenditure is approximately 1000 million Pounds Sterling per year^(19)^. Previous studies have explored outdoor food advertising prevalence around schools^(20–22)^ and/or by measures of neighbourhood deprivation^(23–25)^. However, very little research has been conducted in the UK.

Adams, Ganiti and White (2011)^(26)^ documented the extent of outdoor food advertising by SES in Newcastle, England, and found that 20 % of advertising space and 15 % of all advertisements were for food and just over one-third of food advertisements were deemed ‘less healthy’. The proportion of food advertisements differed across SES, with significantly fewer adverts and less advertising space dedicated to food in the most affluent areas; however, the foods advertised in the most affluent areas were higher in energy density than foods advertised in the least affluent areas. In a more recent study, Olsen *et al.* (2021)^(27)^ explored socio-spatial inequalities in the distribution of unhealthy commodity advertisements in the central belt of Scotland. They found that while unhealthy advertisements (food, alcohol, e-cigarettes or gambling) were unlikely to be found around schools and there was no association between advertisement type and area-level deprivation per se; children in more deprived areas had more engagement with the transport network and therefore were more likely to be exposed to unhealthy advertising overall.

In light of the evidence that food marketing has detrimental effects on health, the WHO recommends that member states restrict children’s exposure to advertising of foods high in fats, trans-fatty acids, sugars, and salt (HFSS) and indicated that places ‘where children gather’ should be free from these forms of advertising^(28)^. In the UK, HFSS food advertising is banned around television programming dedicated to or popular with (based on audience proportion) children aged 4–15 years^(29)^ and a self-regulatory code aims to limit children’s exposure to HFSS marketing online^(30)^. In the summer of 2020, the UK Government announced their intention to strengthen food advertising restrictions on TV and in digital media by the end of 2022 as part of plans to tackle obesity; however, the outdoor advertising of food remains relatively unregulated. Guidance from the UK Advertising Standards Authority (ASA) suggests that HFSS food and non-alcoholic beverage advertising should not be present where 25 % of the audience are under 16 years of age, generally abiding by a 100-m boundary around schools^(31)^. However, there appears to be little, if any, monitoring or enforcement of this guidance and so it is questionable how effective these rules are in practice^(32)^. In February 2019, Transport for London (TFL) implemented a ban on advertising of HFSS foods and non-alcoholic beverages on all forms of public transport across its network to support improvements in public health^(33)^ which may have set a precedent for other regions.

This study aims to quantify the extent of food and beverage advertising on bus shelters in a region of the UK that is yet to introduce any restrictions on this activity, to identify the healthfulness of advertised products and any differences in food advertising by level of deprivation. The study also sought to assess the persuasive creative strategies used and advertisement appeal to children.

## Methods

This study was conducted in South Teesside, specifically the unitary authorities of Middlesbrough and Redcar and Cleveland. South Teesside is a highly deprived region of England. Middlesbrough was ranked as the most deprived of all 343 local authorities in England in 2019, based on the proportion of LSOA (lower super output areas, used for the reporting of small area statistics) being in the most deprived 10 % nationally, and Redcar and Cleveland was ranked 29th^(34)^.

### Data collection

Researchers accessed the location of all bus stops carrying advertising shells across the two local authorities using a National Public Transport Access Node (NaPTAN) code and shelter number, both provided by the local authority’s transport colleagues. Location information was cross-checked against Google Street View (GSV) and then used to identify the latitude and longitude of all bus shelters. A small number (*n* 9) of codes were incorrectly located as some bus shelters had been removed or moved a small distance, since the system was last updated. The impact of this on final results is believed to be negligible as only a small number of shelters were affected.

Two sweeps of data collection were conducted. The first was using GSV in February 2019, where each bus shelter with an advertising shell in the two local authorities was identified and a screenshot taken capturing the advertisement(s) present. Camera images from GSV were dated as June 2018. The second sample of data was collected in March 2019, when a researcher (M.F.) visited each bus shelter with an advertising shell in the sampled areas and took a photograph of the advertisement(s). These images were then recorded against the location of the bus shelter.

Previous research has validated the use of both GSV^(23,25,35)^ and in-person auditing^(18,21,36)^ of outdoor food marketing. The conduct of two sweeps of the advertising at the bus shelters maximises the likelihood that our data are representative of the typical advertising in the sampled areas.

### Coding the data

The coding process was adapted from the WHO TV Monitoring Protocol^(37)^ and the INFORMAS outdoor advertising monitoring protocol^(38)^.

#### Food categories

All bus shelter advertisements were categorised into advert types by one researcher (T.A.), using a modified version of the WHO coding template^(35)^. Food advertisements were then coded as one of seventeen food categories (as per the WHO Regional Office for Europe Nutrient Profile Model (WHO Euro NPM), 2015)^(39)^

#### Nutrient profiling

Nutritional content of advertised food was gathered from the manufacturers’ website or from the food packaging. If this information was no longer available (such as for limited edition fast-food items), nutritional information was gathered from the McCance and Widdowson food database^(40)^ for an item with similar properties (e.g. generic chicken burger). A single researcher (T.A.) classified advertised foods as healthy or less healthy using the UK Nutrient Profiling Model (UK NPM)^(41)^, which is used to determine which items can and cannot be advertised to children on television. Alcoholic beverages were not scored with the UK NPM as they were automatically deemed not appropriate to be advertised to children. The second nutrient profiling was undertaken by a single researcher (A.F) using the WHO Euro Nutrient Profile Model^(37)^ to allow for comparison of the two models and provide a more internationally comparable score.

In accordance with the WHO protocol^(35)^, for brand advertisements, where a brand was shown but no specific product (*n* 8), the biggest selling item sold by that brand was identified using publicly available data and used as a representative product for that brand for the purposes of applying the Nutrient Profile Model. In instances where multiple products were shown, the researcher evaluated which product was the primary focus of the advertisement by considering the placement and size of a product in relation to others in the advertisement in line with previous studies^(42)^.

#### Creative strategies and advertisement appeal

Food advertisements were coded (A.F.) for the presence of persuasive creative strategies, including food images^(43)^, promotional characters (brand equity, licensed or other), celebrity endorsers, competitions, premium offers or reference to social media (e.g. Twitter logo or tag) and whether the advertisement would appeal to children < 18 years. The coding procedure for identifying creative strategies was adapted from the WHO TV Monitoring Protocol^(35)^. The adaptations were to remove variables not relevant to classification of static outdoor advertising images, specifically the use of a musical jingle or characteristic melody, or dynamic audiovisual components.

Appeal to children was judged by considering the advertisement as a whole, similar to previous studies measuring appeal^(33,44)^. The WHO TV Monitoring Protocol states ‘Researcher judgement must be used to decide which groups the ad will appeal to using both visual and verbal cues and knowledge of cultural norms/activities/appeals for young people in the country in question’^(35)^. Therefore, in determining an advertisement to be of appeal to children, we considered the product promoted, creative strategies used (as mentioned above – e.g. if there were characters present, would they appeal to children or not?), alongside broader cues such as appeals to fun, unusual names, child appealing fonts such as bubble writing and bright colours on the advertisement, as these features have been found to appeal to this age group^(45)^.

**Fig. 1:**
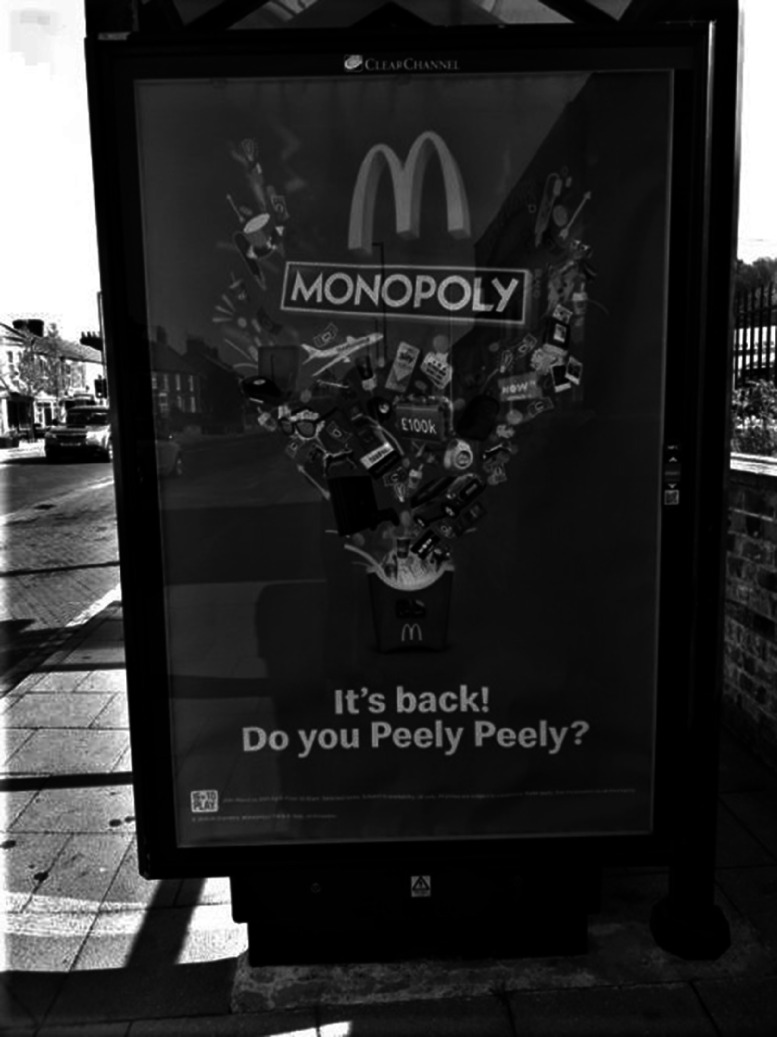
An example of a bus shelter advertisement deemed appealing to children

**Fig. 2:**
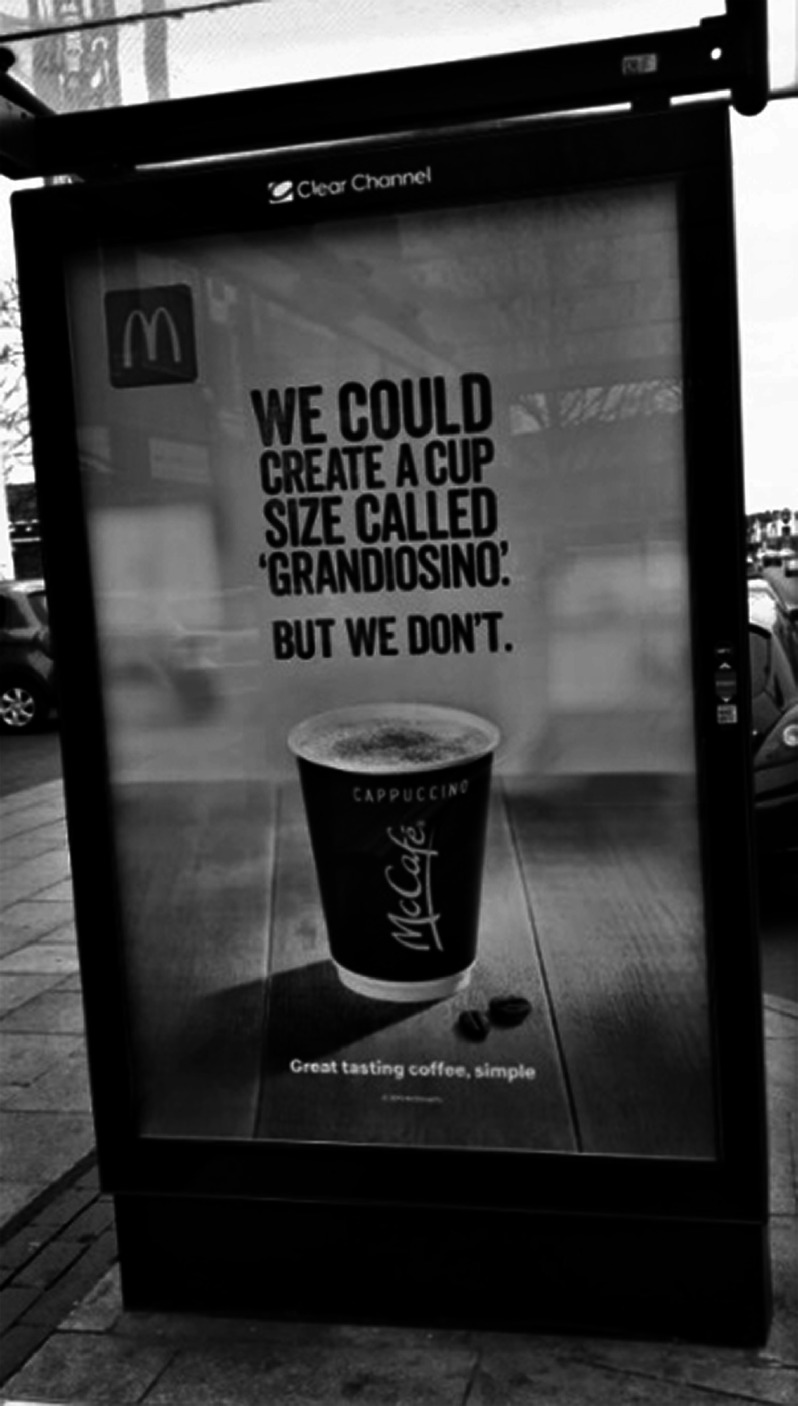
An example of a bus shelter advertisement deemed not appealing to children

Figures 1–2 show examples of advertising deemed appealing to children (Fig. 1) and not appealing to children (Fig. 2).

#### Level of deprivation

The level of deprivation for the location of each bus shelter advertisement was identified using latitude and longitude coordinates checked against the Government Index of Multiple Deprivation database^(32)^. The IMD ranks small geographical areas in the UK on seven indices: income, employment, health deprivation and disability, education, crime, barriers to housing and services, and living environment. This index gives each neighbourhood within each authority an IMD decile rating of 1–10, 1 being the most deprived and 10 being the least deprived.

#### Inter-rater reliability

All bus shelter advertisements were coded by one researcher and 10 % were also coded by the second researcher. Estimated agreement was 0·965 with a kappa score of 0·859. As this exceeded the 0·8 threshold, inter-rater reliability was deemed near perfect.

Another researcher categorised foods into the 17 WHO Euro NPM^(37)^ categories, recorded the nutritional information for every advertised food or non-alcoholic beverage product and used the UK NPM to determine whether the advert was classed as healthy or less healthy. The second researcher coded 10 % of these, with an estimated agreement of 0·89 and a kappa score of 0·73. As the estimated agreement again surpassed the 0·8 threshold, inter-rater reliability was deemed substantial.

### Statistical analysis

Alongside descriptive statistics of food advertising across the areas assessed provided as percentages, a chi-square test was conducted (using IBM SPSS Statistics for Windows, version 26.0. IBM Corp.) to determine whether there was a significant difference in the proportion of healthy/less healthy advertisements by IMD decile. For this analysis, deciles were grouped into high deprivation (1–3), medium deprivation (4–7) and low deprivation (8–10). We considered results significant at *P* < 0·05.

## Results

A total of 832 bus shelter advertisements were identified overall (from the two samples, GSV and in person, combined). Almost half (48·9 %) of these were for foods and beverages (including alcohol), 44·5 % (*n* 370) of all advertisements were for food and non-alcoholic beverages (Table 1). Remaining analyses referring to foods are exclusive of alcoholic beverages. Just 2·2 % of these advertisements (*n* 8) were promoting a brand only, all others (*n* 362) were promoting a product.

**Table 1:** Proportion of each advertisement type across Middlesbrough and Redcar and Cleveland

	Proportion of each advertisement type		
	Middlesbrough % (*n* 529)	Redcar and Cleveland % (*n* 303)	Total % (*n* 832)
Food/non-alcoholic beverage	46·3	41·3	44·5
Entertainment	18·7	28·7	22·4
Other	9·2	7·6	8·5
Financial	3·6	9·2	5·6
Utilities	6·2	2·3	4·8
Alcoholic beverage	5·5	2·6	4·4
Channel promotions	3·6	4	3·7
Clothes/shoes	4·2	0·7	2·9
Public information announcements	2·3	3	2·5
No advertisement^*^	0·6	0·7	0·6

* No advertisement refers to when the advertising shell was left without a poster.

In Middlesbrough, there were 529 advertisements of which food advertisements comprised 46·3 % of the sample. In Redcar and Cleveland, there were 303 advertisements, of which 125 (41·3 %) were for food. The second most prevalent advert type across both authorities was entertainment, with a total of 186 (22·4 %) advertisements, followed by the ‘other’ advertisement category (8·5 %) which was comprised of adverts for travel, toiletries, household equipment and cleaners, charities, national lottery, gambling, local events, search engines and fitness (gyms) among some other categories. 5·6 % of advertisements were financial. All other advertisement types took up less than 5 % of the advertising space on bus shelters. Thirty-seven advertisements (4·4 %) were for alcoholic beverages. The breakdown of all advertisement types is shown in Table 1.

Just over a third (33·3 %) of food advertisements were ‘other beverages’ (Table 2) – which includes all sugar-sweetened beverages (SSB) as well as sugar-free beverages and water. Some SSB were also present in the ‘beverages (milk)’ category, which took up 6·8 % of all food advertisements. Within the sample, there were a total of 131 adverts for SSB (35·5 % of all food advertisements) and 10 adverts for sugar-free beverages (2·7 % of all food advertisements). 24·1 % of food advertisements were for ready-made/convenience foods, and this included products such as burgers and sandwiches. Over a fifth (21·1 %) of food advertisements were classed as processed fruit, vegetables and legumes, all of which promoted the McDonalds brand. Almost all of these advertisements (75/78) had an image of the container for fries (a processed vegetable) at the forefront of the advertisement. For the remaining three advertisements promoting the McDonalds brand, French fries were selected as a representative product of the brand (for the purposes of nutrient profiling), as the biggest selling item^(46)^.

**Table 2:** Proportion of food advertisements in each WHO food category across the total sample

WHO food category	Proportion of food advertisements
Beverages – other	33·3
Ready-made/convenience food	24·1
Processed fruit, vegetables and legumes	21·1
Beverages – milk drinks	6·8
Cakes, sweet biscuits and pastries	3·8
Savoury snacks	3·0
Butter and other fats/oils	2·7
Edible ices	1·9
Chocolate and sugar confectionary	1·9
Processed meat, poultry or fish	0·5
Yogurts	0·5
Cheese	0·3

Just under a third (30·6 %) of all advertisements and 62·7 % of all food advertisements in the sample promoted McDonalds. All other food brands each took up less than 4 % of advertising space. It is notable that overall, the second most frequent brand encountered was Desperados, advertising beer, with twenty-five total adverts (6·1 %).

Overall, 130 (35·1 %) food advertisements were classed as ‘less healthy’ (Table 3) by the UK NPM and therefore not suitable to be advertised to children. Just over a third (33·9 %) of food advertisements in Middlesbrough were classed as less healthy, for Redcar and Cleveland it was 37·6 % (Table 3). For comparison, using the WHO Euro NPM, 79·5 % (*n* 294) of food advertisements were classed as not permitted to be advertised. This made up 83·7 % of food advertisements in Middlesbrough and 71·2 % of food advertisements in Redcar and Cleveland.

**Table 3:** Proportions of less healthy and healthy advertised foods in each WHO Euro NPM category

	% in Middlesbrough	% in Redcar and Cleveland	% Total
Proportion of food ads classed as healthy (UK NPM)	66·1	62·4	64·9
Proportion of food ads classed as less healthy (UK NPM)	33·9	37·6	35·1
Proportion of food ads permitted (WHO Euro NPM)	16·3	28·8	20·5
Proportion of food ads not permitted (WHO Euro NPM)	83·7	71·2	79·5
Ads in each category as a proportion of all less healthy ads:			
Ready-made/convenience foods	19·3	34	24·6
Beverages – milk drinks	27·7	4·3	19·2
Beverages – other	15·7	17	16·2
Cakes, sweet biscuits and pastries	12	8·5	10·8
Savoury snacks	9·6	6·4	8·5
Butter and other fats/oils	3·6	14·9	7·7
Edible ices	4·8	6·4	5·4
Chocolate and sugar confectionary	3·6	8·5	5·4
Processed meat, poultry or fish	2·4	0	1·5
Cheese	1·3	0	0·7
Ads in each category as a proportion of healthy ads:			
Beverages – other	36·4	56·4	42·9
Processed fruit vegetables and legumes	30·9	35·9	32·5
Ready-made convenience food	32·1	6·4	23·8
Yogurts	0·6	1·3	0·8

Of food products classed as less healthy by the UK NPM (Table 3), the most frequent food category, making up just under a quarter (24·6 %, *n* 32) of these advertisements, were for ready-made/convenience foods, followed by milk beverages (19·2 %) and other beverages (16·2 %). Redcar and Cleveland have over four times the proportion of advertisements for butter and other fats/oils as well as more than double the proportion of advertisements for chocolate and sugar confectionary compared with Middlesbrough, although these differences were not notable when comparing by frequency of advertisements (*n* 7 *v*. *n* 3 and *n* 4 *v*. *n* 3, respectively). Meanwhile, Middlesbrough had over six times the proportion of advertisements for milk beverages than were found in Redcar and Cleveland (*n* 23 *v*. *n* 2).

The proportion of food types advertised that were classed as healthy are also shown in Table 3. There were no advertisements for some food categories including fresh or dried pasta, rice or grains, fresh and frozen fruit, vegetables or legumes, and fresh or frozen meat, poultry or fish.

From Table 4, it can be seen that almost all food advertisements (98·9 %, *n* 366) used one of the persuasive creative strategies we assessed as likely to increase advertisement appeal (98·8 % in Middlesbrough and 99·2 % in Redcar and Cleveland). Over three-quarters (79·5 %, *n* 291) of food advertisements used a food image to promote the product. About a fifth (20·5 %, *n* 75) promoted a competition, specifically ‘McDonalds Monopoly’. The use of characters was rare, with just 0·5 % (*n* 2) of food advertisements featuring licensed characters and 1·6 % (*n* 6) including ‘other’ characters (namely cartoon people (*n* 5) and a cartoon animal (*n* 1)). Eight advertisements used more than one of these strategies, and four used none at all. A majority of food advertisements (71·9 %, *n* 266) were considered to appeal to children under 18 years, 38·7 % of which were classed as ‘less healthy’.

**Table 4:** Proportion of food advertisements using persuasive creative strategies

	Proportion of food ads using creative strategies (total %)	Middlesbrough %	Redcar and Cleveland %
Food ads using any persuasive creative strategies	98·9	98·8	99·2
Food image	79·5	79·2	77·6
Competition	20·5	19·6	21·6
Other character	1·6	0·8	3·2
Licensed character	0·5	0·4	0·8
Brand equity character	0·0	0·0	0·0
Celebrity endorser	0·0	0·0	0·0
Social media	0·0	0·0	0·0
Premium offer	0·0	0·0	0·0

**Fig. 3:**
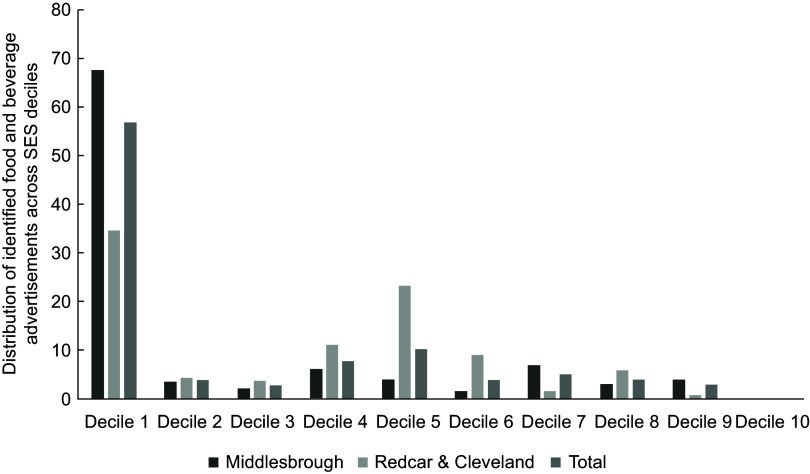
A clustered bar chart showing the spread of food and beverage advertisements on bus shelters in Middlesbrough and Redcar and Cleveland across English Index of Multiple Deprivation (IMD) deciles. IMD deciles are the official measure of relative deprivation for small areas in England – 1 = highest deprivation, 10 = lowest deprivation

**Fig. 4:**
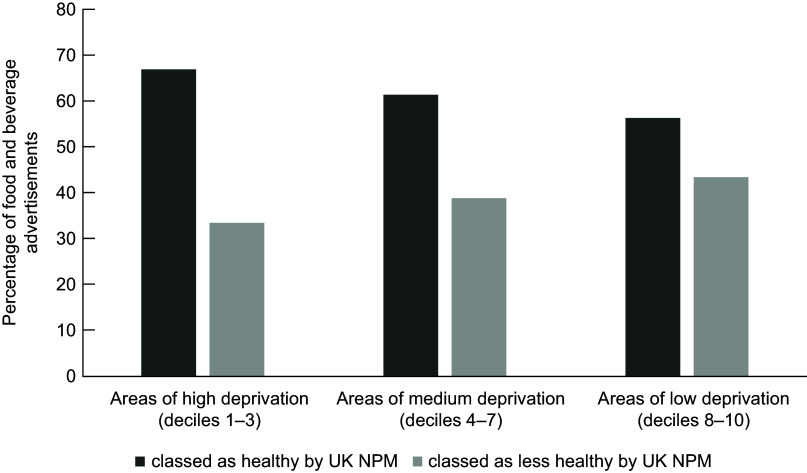
A clustered bar chart showing the proportion of healthy/less healthy foods across low, medium and high deprivation areas. IMD deciles have been grouped into high deprivation (deciles 1–3), medium deprivation (deciles 4–7) and low deprivation (deciles 8–10)

Of the food advertisements, 58·4 % were in the most deprived decile (Decile 1) largely driven by the concentration of these adverts in the town centre (Decile 1 wards) in Middlesbrough (see Fig. 3). The second most frequent decile for food advertisements was Decile 5. No food advertisements were present in Decile 10 (the least deprived). This is representative of the proportion of bus shelters in each Decile, with 51·8 % of all bus shelters being in Decile 1 areas (*n* 313) and 0·5 % in Decile 10 (*n* 3). No significant differences were found in the distribution of healthy/less healthy food marketing across deciles (*X* ^2^(2, *n* 362) = 1·658, *P* = 0·437, see Fig. 4).

## Discussion

This study quantified the extent and nature of food and beverage advertising on bus shelters in a deprived area of the UK. Almost half of all advertisements (48·9 %) on bus shelters were for food and beverages, and the next most frequent advert type was entertainment (22·4 %). This is a higher prevalence of food advertisements than was found in previous UK studies of outdoor advertising (15 %)^(24)^, television (12·8 %)^(40)^ and on bus shelters in New Zealand (25·5 %)^(23)^. This illustrates that bus shelters are a key platform for food marketing, and warrant consideration by researchers seeking to increase understanding of the impact of different forms of marketing on eating behaviour and by policymakers considering restrictions on food marketing as part of public health policies to tackle obesity. Differences in relative prevalence of food advertising between the present study and that of Adams *et al.* (2011)^(24)^ may reflect the types of outdoor advertisements measured (bus shelters *v*. all outdoor, including billboards), and differences with Huang *et al.* (2020)^(23)^ may be at least partly a result of the areas searched. Huang *et al.* included bus shelters up to 500 m around schools, whereas this current study included all bus shelters with advertisements across two local authorities and did not focus on school locations. It is possible that brands are more mindful of the advertisements placed around schools because of self-regulatory advertising codes^(47)^.

Of the food advertisements, a substantial proportion (35·5 %) were for SSB. This is problematic, as consumption of SSB has been found to be positively associated with weight gain and obesity in both children and adults^(48)^. If a large proportion of what consumers are exposed to through outdoor advertising are SSB, consumption levels may be impacted (especially in children and young people) and therefore health of the population. There is little research on direct impacts of outdoor food advertising; however, Lesser, Zimmerman & Cohen (2013)^(49)^ found that for every 10 % increase in exposure to outdoor food advertising, participants consumed on average 6 % more soda, equating to an additional 196 kilocalories (820 kilojoules) a week. Another study conducted across the UK, USA, Canada, Mexico and Australia^(50)^ found that outdoor marketing of SSB in the ‘functional environment’ was significantly associated with a likelihood of high SSB consumption compared with no SSB consumption. This relationship was consistent across the five countries studied. More research on the impact of outdoor food advertising is needed to support these findings and inform policy.

At a brand level, the pervasiveness of McDonalds marketing is perhaps expected, given that the corporation spent 476·8 million USD on advertising worldwide in 2018 and 447·3 million USD in 2019^(51)^. In the UK alone, their advertising expenditure rose from £56 million in 2012 to £100 million in 2016^(52)^. Fast food is also consistently one of the most advertised product categories in monitoring research^(40,53)^. However, a large proportion of McDonalds adverts were not for less healthy foods, instead promoting the brand itself or products classed as healthy by the UK NPM such as coffee and French fries. Future research should examine the influence of brand advertising to identify whether mere exposure to the brand may lead to unhealthy behaviours.

Nutritional content of foods advertised were obtained and scored primarily using the UK NPM^(39)^, although for a comparison and to facilitate international comparability, the WHO Euro NPM^(37)^ was also used. Foods classified as less healthy by the UK NPM cannot be advertised on television in the UK during children’s viewing times; however, when it comes to outdoor advertising, there is only ‘guidance’ suggesting these products should not be advertised within 100 m of school boundaries – additionally, this ‘guidance’ is vague regarding how 100 m should be measured (e.g. 100 m as the crow flies or 100 m walking distance?). In total, 35·1 % of food advertisements were for less healthy products (15·6 % of all bus shelter advertisements), and therefore can be said to be not suitable to be advertised to children; however when using the WHO Euro NPM, 79·5 % of food advertisements were not permitted to be advertised.

Previous research by Patiño *et al.* (2016)^(54)^ found that a higher proportion of products were classed as excessive in undesirable nutrients by the WHO Euro NPM (83·1 %) than the UK NPM (78·7 %), and both of these rated more foods as undesirable than the Mexican Ministry of Health Nutrient Profile Model, which indicated that 64·3 % of foods did not comply with Mexican nutritional standards. This latter finding was largely explained by greater allowance of energy, sugar and fat in the Mexican model. Differences between the WHO Euro and UK NPM can be put down to the WHO Euro considering only aspects of concern such as excess fat, saturated fat, sugar, sweetener, salt and energy. The WHO Euro NPM does not permit beverages with any added sugar or sweetener, and products such as McDonalds fries and the Chicken Legend were classed as not permitted due to exceeding thresholds for fat, and salt and energy (kcal), respectively. In comparison, the UK NPM does not have limits regarding added sweetener and it allows a small amount of added sugar; therefore, diet soft drinks were classed as healthy. A score calculated using the UK NPM also considers the healthy nutrients in a product (e.g. protein, vegetables and fibre) and offsets these against the undesirable nutrients, which can lead to some classifications that appear counterintuitive (such as McDonalds fries being healthy). Foods not permitted by the WHO Euro NPM have consistently made up a majority of food advertisements outdoors, with previous studies reporting 50·2 %^(23)^ and 89·2 %^(55)^, and, in the present study, 79·5 %.

This study sought to identify the persuasive creative strategies used in bus shelter food advertising and the extent to which the advertising might appeal to young people. Following adaptations to the WHO TV Monitoring Protocol^(35)^, and consideration of outdoor-specific content within the INFORMAS protocol^(36)^, the remaining strategies relevant for classification were consistent with those found to be most prevalent in TV food marketing to children^(10)^. Almost all food advertisements (98·9 %) used at least one of the pre-defined strategies. The majority of food advertisements (79·5 %) featured an image of food, which have been shown to have a powerful impact on craving, eating behaviour and body weight as impactful as real food exposure and more impactful than olfactory cues^(56)^. Over a fifth promoted a competition, all of which were for McDonalds Monopoly. It was deemed that 71·9 % of food and non-alcoholic beverage advertisements would appeal to children under 18 years of age and of these adverts 38·7 % were classed as ‘less healthy’ by the UK NPM. Appeal is subjective, and a limitation of this study is that the coding protocol was adapted largely from one designed for audiovisual advertising content and operational definitions were not established to identify child appeal. The development and validation of a comprehensive protocol specific to outdoor food advertising that addresses these issues is a priority, to support the conduct of a robust and comparable monitoring research. However, the characteristics of advertisements considered when making this judgement (e.g. premium offers, promotional characters and appeals to fun) were based on the extensive literature on the nature of child-directed marketing^(57)^, and inter-rater reliability was high for our judgements of appeal, so these results are likely a reliable representation of child appealing outdoor advertising.

The detrimental effects of unhealthy food marketing exposure via other avenues, most notably television and the internet, on eating and eating-related outcomes in young people are well documented^(6,58)^. Advances in bus shelter advertising have enabled an increase in digital advertisements, with one outdoor advertising company reporting a network of more than 2400 digital bus shelters across 136 cities and towns in the UK^(59)^. Although no digital bus shelters were found in this study, as the number of these continues to increase, the line between digital and outdoor marketing will become increasingly blurred which is a challenge for public health research and policy alike.

There was no clear trend between the proportion of food and beverage advertisements or healthiness of food advertisements across IMD Deciles, and a chi-square test found no significant differences in healthiness of foods advertised by level of deprivation. This null finding may be a result of both Middlesbrough and Redcar and Cleveland being highly deprived areas, and it is possible that there was not enough variation in IMD index for the bus shelters in this sample to accurately assess inequalities. There was a large proportion of food and beverage advertisements in Decile 1 (most deprived) and no food and beverage advertisements in Decile 10 (least deprived), but this latter finding was likely due to there being very few (*n* 3) bus shelters with advertisements in Decile 10 areas. Low-decile areas mostly covered the town centres which are often the most deprived parts of a city^(60)^ and generally have the best transport links and therefore higher number of bus shelters. It is possible that the high proportion of food advertisements in Decile 1 is reflective of this. Given that both Middlesbrough and Redcar and Cleveland are some of the most deprived areas of the UK^(32)^, it is not unexpected that Decile 1 areas make up the largest geographical area, and so contain the largest number of bus shelters across the regions.

Future studies should seek to include sufficient samples from each decile to permit comparisons of this kind. While this study captured advertisements over two time points, it still does not support longitudinal evaluations. There is also no measure of population awareness of, attention to, or response to the advertising content, and therefore without further research it is not possible to draw firm conclusions as to the extent to which these advertisements are influencing behavioural outcomes.

This study shows the extent of unhealthy food advertising on bus shelters in a deprived area of the UK. Although there did not appear to be evidence of social patterning of bus shelter food advertisements in this sample, it may be expected that those of lower SES would be more likely to use the public transport network and therefore the findings of this study suggest bus shelters, alongside TV^(61)^ are contributing to inequalities in exposure to unhealthy commodity advertising^(25)^. Future research should seek to quantify the impact of exposure to outdoor food advertising upon food behaviours (such as choice and intake) in order to inform public health strategies to curb obesity.

The UK has one of the highest rates of obesity (both child and adult) in the world, but positive steps are being taken to reverse this. National policy recommendations are proposing to limit the population exposure to advertising of foods that are high in fat, salt and sugar (as determined by the UK NPM) on television and online. The responsibility for the provision of actual bus shelters is a local one (via Local, Combined or Transport Authorities) and most contracts currently only restrict a limited range of items from being advertised (e.g. tobacco, politics and religion). The work presented here shows that advertising in bus shelters includes a large proportion of food and drink products that would otherwise be banned under current television restrictions.

There are a number of reasons why making changes to bus shelter contracts and the associated advertising might be difficult. For example:

- The advertising which is part of bus shelter contracts represents a means of reducing expenditure for local authorities as the income offsets the cost of providing the actual infrastructure for the provider. In some high population areas, it may represent actual income to the local authority. By restricting food and drink advertising, local authorities may assume that they will be limiting the income coming into the contract. However, previous work suggests not – when the advertising of food and drink was restricted on children’s television, other marketing campaigns took up this space and more recently, the TfL ban has not resulted in any loss of income (in fact, overall income rose by £2·3 million between 2018/19 and 2019/20, to £158·3 million^(62)^ (Transport for London Advertising Report, 2020). As other work progresses (e.g. London Borough of Southwark), additional case studies will emerge.
- Many of the bus shelter contracts are long term because of the amount of capital that a provider has to invest at the outset – hence they are often 10- or 15-year contracts. Therefore, some areas may not be in a position to make changes contractually in the short term (at least not without national legislative change). The Child Obesity Trailblazer Programme^(63)^ in Lewisham is working with their current provider to look to make changes mid-contract, with positive initial findings which provides a case study for other local authorities to follow.
- There are only a limited number of providers of bus shelter infrastructure – for example, Clear Channel, Trueform and JCDecaux. These may be resistant to change; however, the case study examples provided above (London Borough of Southwark, Child Obesity Trailblazer in Lewisham) shows that such change is possible.

Therefore, the changes to bus shelter food marketing that are needed are slowly becoming conceivable. With the potential to overcome concerns regarding bus shelter contracts and loss of income, the examples mentioned have paved the way for other local authorities to follow suit with a reduced likelihood of risk. Local authorities are THE champion of local action^(64)^ and have had the responsibility for public health since 2013. Many are adopting whole systems approaches to obesity reduction. Reducing food and drink advertising must be considered as part of these approaches, especially as part of a wider approach to advertising and public health similar to the approach of the London Borough of Southwark and others.

In addition to the level of food and drink advertising found in the samples presented here, there was also a worrying amount of advertising for alcohol. Hence, this report and the approaches proposed within should be of interest to other colleagues and regional/national partners (e.g. Balance, Public Health England Alcohol, and Drugs and Tobacco team).

## Conclusion

There is little previous research conducted into outdoor food marketing in the UK. This study presents the extent of food advertising on bus shelters in a deprived area of the UK, illustrating the pervasiveness of food advertising, and the substantial proportion of foods classed as less healthy by the UK NPM. These less healthy products are not permitted to be advertised on children’s TV, yet children can still be frequently exposed when outdoors, to unhealthy advertising that is appealing to them. Fast-food brand McDonalds were responsible for the majority of food advertisements (62·7 %), although many of the promoted items such as coffee and fries were classed as healthy. Future research should seek to elucidate the impact of outdoor food marketing, and in particular the impact of brand rather than product advertising, to find whether there is a demonstrable relationship with eating behaviours and health outcomes.
